# Atraumatic Transdiaphragmatic Intercostal Hernia Following a Coughing Spell: A Case Report

**DOI:** 10.7759/cureus.107498

**Published:** 2026-04-21

**Authors:** Marc R Mohammed, Jessica Piazza, Tara Ranjbar, Rohit Shahani, Manuel Villa Sanchez

**Affiliations:** 1 Medicine, Touro College of Osteopathic Medicine, New York, USA; 2 General Surgery, Staten Island University Hospital, Staten Island, USA; 3 Thoracic Surgery, Staten Island University Hospital, Staten Island, USA

**Keywords:** cardiothoracic surgery, copd, diaphragmatic hernia, intercostal rupture, large intestine herniation, liver herniation, thoracic hernia, transdiaphragmatic intercostal hernia

## Abstract

Transdiaphragmatic intercostal herniations are rare clinical entities that require precise management. Although risk factors are not well established, these defects do occur in clinical practice. Of the several reported cases of transdiaphragmatic intercostal hernias, few have involved the liver and large bowel. The present case illustrates a rare finding of a 60-year-old male patient who presented to the emergency department experiencing severe pain and shortness of breath after feeling a “pop” on the right side of his chest following a violent episode of coughing that had been ongoing for a couple of days. Imaging revealed a right seventh rib fracture with herniation and incarceration of the liver and large bowel within the intercostal space. Our case highlights the importance of diagnostic imaging, clinical management, and surgical considerations when managing patients with this rare surgical entity.

## Introduction

Transdiaphragmatic intercostal herniation (TDIH) was first reported in 1946, and to date, only 42 cases have been documented [1,2]. Due to the rarity of TDIH, there is still no consensus regarding its management and clinicians are left to individually tailor their patients’ management [3]. Although there are limited reports of these chest wall injuries, most cases occur after trauma to the chest wall or surgical intervention. However, they may also spontaneously occur from any condition that increases intra-abdominal pressure [3,4]. These structural defects are further classified as congenital or acquired [3]. TDIHs differ from intercostal abdominal hernias in that the former involve a diaphragmatic defect associated with the herniation [5]. With a skilled medical and surgical team, prompt diagnosis and treatment are crucial to prevent complications such as bowel obstruction, ischemia, or respiratory compromise.

Although clinical signs may suggest herniation through the chest wall, a contrast-enhanced computed tomography (CECT) scan is the gold standard for confirming the diagnosis. This scan allows for the evaluation of the contents of the hernia sac, exclusion of other pathologies, and formulation of an appropriate surgical plan to correct any defects in a timely manner [4].

Our case report discusses the surgical management of a 60-year-old male patient who presented to the emergency department with painful worsening dyspnea and increasing oxygen requirements despite supplemental oxygen via nasal cannula. His CT scan confirmed a TDIH that demanded urgent surgical intervention.

## Case presentation

A 60-year-old male patient with a past medical history of hypertension, chronic atrial fibrillation, chronic obstructive pulmonary disease (COPD) on home oxygen, recurrent pneumonia, obesity, and a 50-pack-year smoking history presented to the emergency room with worsening shortness of breath after feeling a “pop” on the right side of his chest following an uncontrolled coughing spell. His vital signs were stable while receiving 2 L/min of oxygen via nasal cannula, and he had right chest wall tenderness with a palpable right thoracic herniation, without overlying ecchymosis or erythema. His white blood cell (WBC) count was elevated to 14.86 x 10^9^/L, and the remainder of his laboratory studies were unremarkable (Table 1).

**Table 1:** Initial laboratory values

Parameter	Value	Reference Range
White Blood Cell Count	14.86 x 10^9^/L	4.5–11.0 x 10^9^/L
Hemoglobin	13.4 g/dL	13.5–17.5 g/dL (male)
Hematocrit	39.90%	41–53% (male)

**Figure 1:**
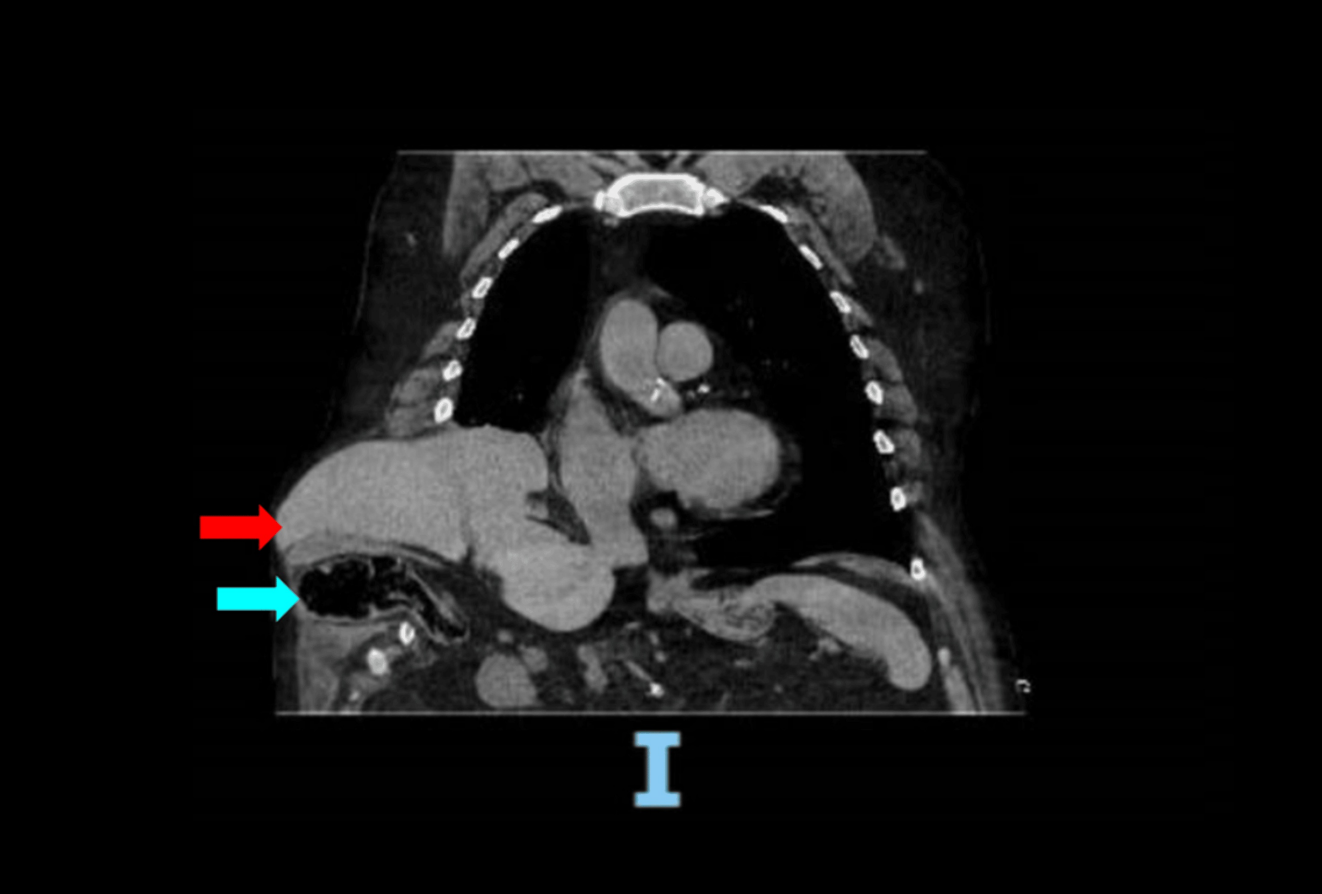
Coronal computed tomography image showing increased herniation of the right hepatic lobe and new herniation of nonobstructed right colon through the right seventh intercostal space. Red arrow: Right lobe of the liver; Blue arrow: Large bowel segment.

A CT scan showed an acute, mildly displaced posterolateral right seventh rib fracture with herniation of the liver and colon into the intercostal space (Figure 1).

**Figure 2:**
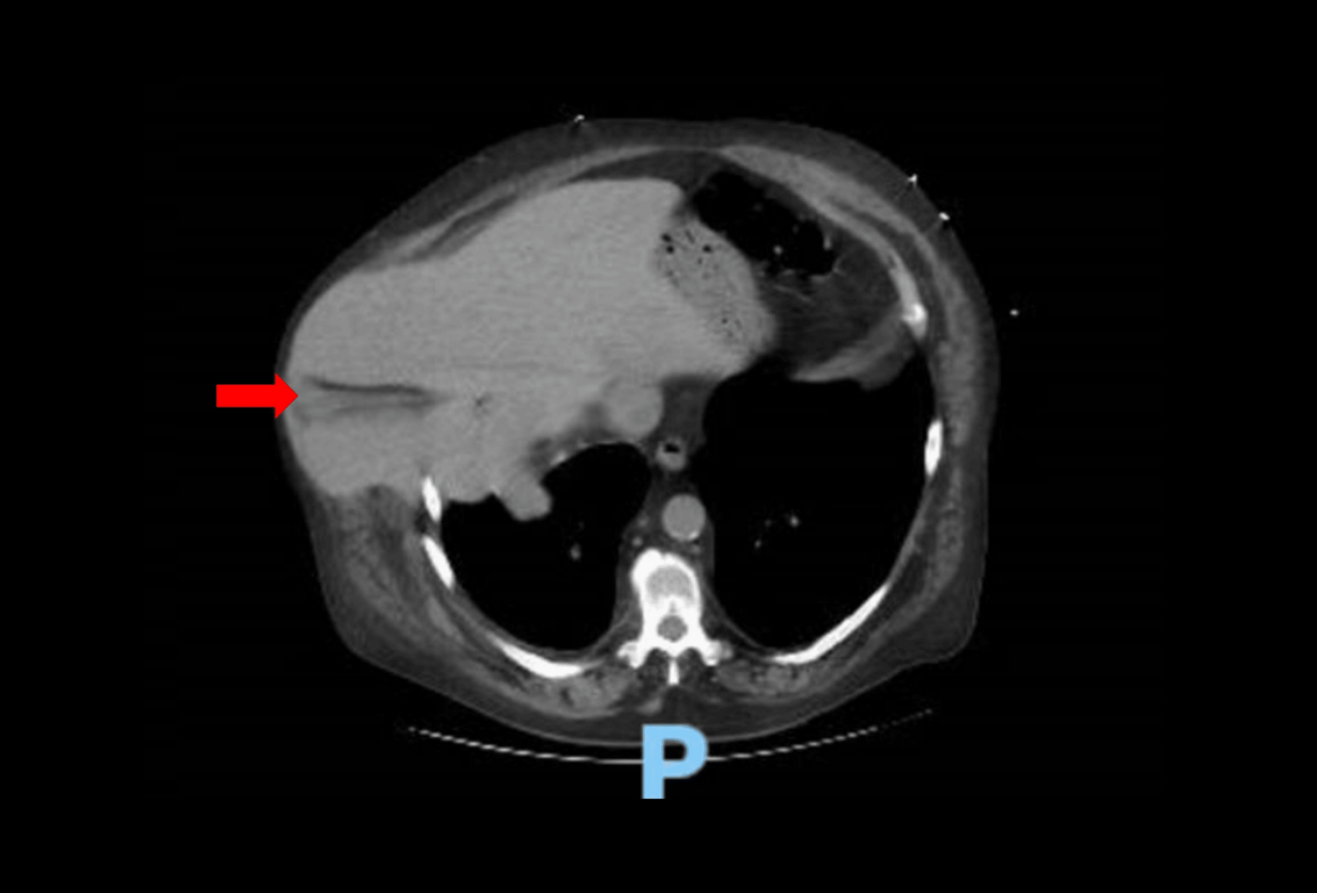
Axial computed tomography image showing right liver lobe herniation Red arrow: Liver herniation

The patient was urgently taken to the operating room for a combined thoracoabdominal approach for this complex surgical condition. The decision to use a combined thoracoabdominal approach was driven by the need to safely reduce the incarcerated organs from below and to adequately reconstruct the complex chest wall and diaphragmatic defects from above. During robotic-assisted laparoscopy, a large diaphragmatic hernia with extensive adhesions was seen, with at least 50% of the liver and the hepatic flexure of the colon within the hernia sac into the chest wall and the pleural space (Figure 2).

**Figure 3:**
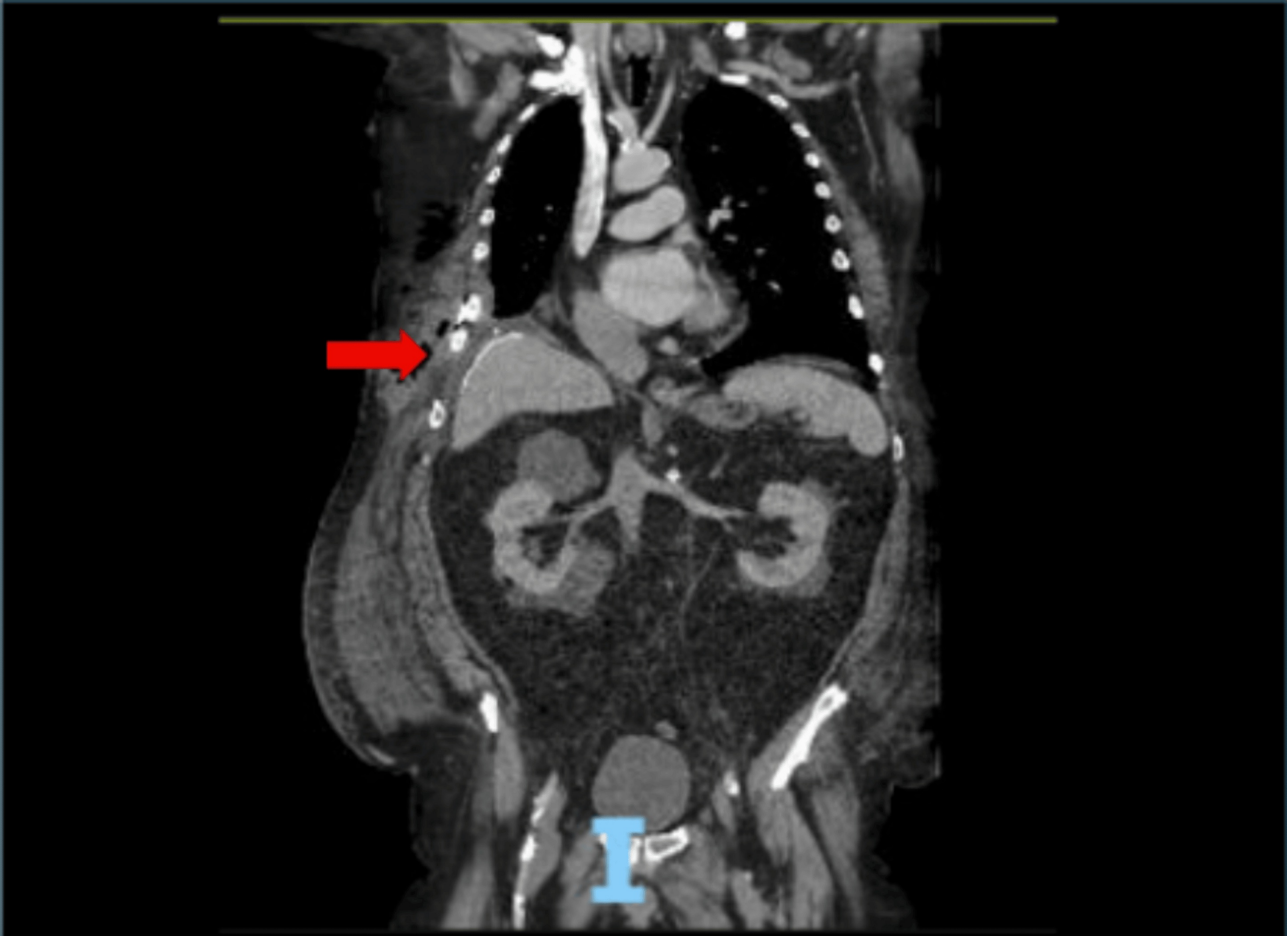
Coronal computed tomography image displaying diaphragmatic hernia repair with mesh, chest wall hernia repair, and approximation of the rib fractures Red arrow: Area of rib plating and diaphragmatic mesh repair.

**Figure 4:**
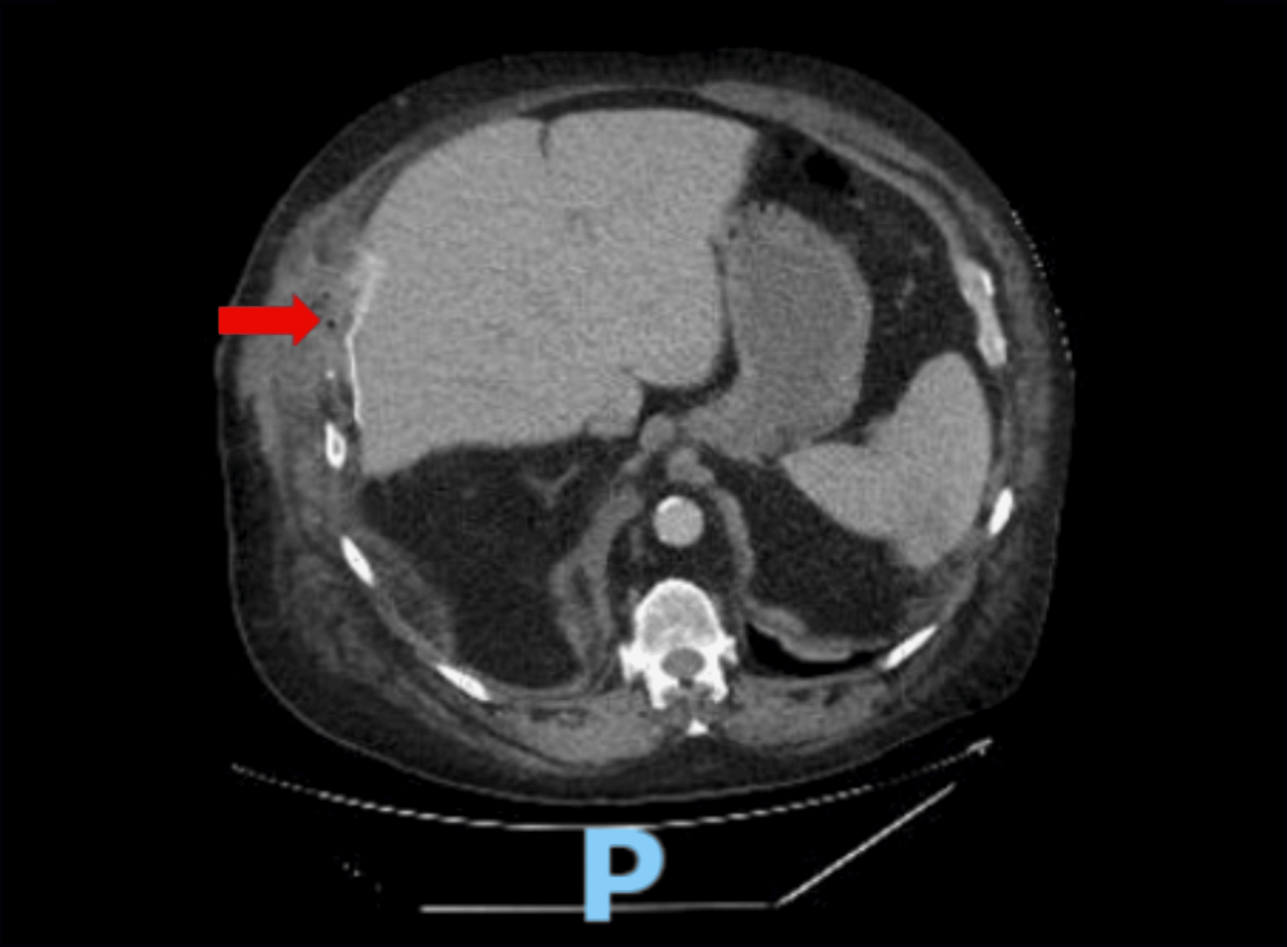
Axial computed tomography image showing surgical repair of the liver herniation Red arrow: Area of diaphragmatic mesh repair.

A right thoracotomy incision at the ninth intercostal space revealed disruption of the costal margin with the rib fracture and a large diaphragmatic defect. To close the intercostal hernia, the ninth and 10th ribs required three plates each placed posteriorly, while the cartilage was approximated anteriorly with three figure-of-eight fibrous tape sutures (Figures 3, 4).

Rib plating was deemed necessary not only for fracture fixation but also to provide rigid stabilization and prevent recurrence. The diaphragmatic hernia was repaired with a dual-layer GORE® DUALMESH® Biomaterial (W. L. Gore & Associates, Inc., Flagstaff, AZ, USA), secured in place with size zero ETHIBOND® braided polyester sutures (Ethicon, Inc., Somerville, NJ, USA). Cryoablation nerve block was performed from T4-T8 for postoperative pain control.

Postoperatively, the patient did well following an early extubation and was then transferred from the intensive care unit (ICU) overnight to the telemetry floor. The patient's home medication regimen was continued without changes, including corticosteroids and bronchodilators. On postoperative days three and four, the patient was seen by physical therapy and ambulated 100 feet on two separate encounters, and was recommended outpatient physical therapy. A 28 Fr chest tube remained in place for a week, given the patient’s advanced COPD, and the patient was discharged uneventfully a couple of days later. Postoperative outpatient follow-up showed a stable and improving chest X-ray. 

## Discussion

This patient presented with worsening dyspnea and desaturation following a self-reported episode of violent coughing, causing increased intra-abdominal pressure and herniation of the right lobe of the liver and large bowel. In a 2021 case report and literature review of 42 reported TDIHs, Ioannidis et al. identified four cases involving the liver and large bowel [1]. TDIHs are rare clinical entities that require a strategic approach to restore physiological function and prevent potential complications. While most cases occur after traumatic injury to the chest wall, some cases have been reported following coughing episodes with rib fractures, particularly in patients with underlying conditions such as COPD, asthma, osteoporosis, or advanced age [4]. Our patient, who had advanced COPD, experienced this unfortunate event.

Various imaging modalities, including chest X-ray, CT scan, magnetic resonance imaging, and ultrasound, can be utilized to make this rare diagnosis. According to Chaturvedi et al., CT imaging is the modality of choice in identifying and characterizing thoracic hernias in adults. CT allows for the measurement of hernia and sac dimensions, analyzing hernia contents, and is useful in assessing any accompanying complications and for surgical planning [6].

Surgery is the only definitive intervention to treat TDIH to avoid complications such as strangulation and tissue necrosis. The location and degree of herniation in the chest wall are determining factors in the management of the hernia. Although the general management for chest wall hernias in supraclavicular or subscapular areas is conservative management, anterior chest wall hernias require surgical management regardless of symptoms [7]. Anterior chest wall hernias are at risk of extension into the abdominal wall, creating a thoracoabdominal herniation, as seen in our case. Thoracoabdominal herniations have increased morbidity due to complications involving the herniated contents as well as the increased complexity of the repair [7]. Due to the rarity of traumatic chest wall hernias, there are few guidelines outlining the severity of the injury and the appropriate surgical management of the injury. The specific surgical management for a chest wall herniation is case dependent and varies in the use of mesh, technique, pre-operative management and antibiotic use.

Utilizing classification systems for complex hernias may help guide treatment strategies. Although currently there are no classification systems relating to spontaneous chest wall hernias involving abdominal contents, Kuckelman et al. developed a grading system for traumatic chest wall herniation based on the extent of damage, location and size, and the appropriate surgical management for the graded hernia. This system, termed Traumatic Rib Cage Hernias (TRCH), is classified from Grade one to Grade five. Grade one is defined as an intercostal muscle tear less than 2 cm in width, fewer than two simple nondisplaced adjacent rib fractures, and no or minimal organ herniation. Grade two consists of multiple or displaced rib fractures, an intercostal muscle tear greater than 2 cm in width, with partial organ herniation. Grade three involves multiple comminuted rib fractures, loss of portions of ribs, and organ herniation. Grade four represents Grade three injuries with additional disruption of the overlying chest and/or abdominal wall musculature and organ herniation. Finally, Grade five is characterized by an open defect with full-thickness loss of the chest wall, organ herniation, and marked loss of hemithorax domain [8]. Although designed for traumatic cases, this classification system may offer a useful framework for spontaneous chest wall hernias. Our patient’s presentation most closely resembled a Grade five injury, and management followed principles used in high-grade traumatic cases. Further research is needed to clarify its role in spontaneous chest wall hernia cases. The proposed surgical repair for a Grade five chest wall herniation includes debridement, rib plating, and prosthetic mesh reinforced soft tissue repair [8]. Our case required rib plating due to the complete disruption of the costal margin, and GORE® DUALMESH® for the diaphragmatic hernia repair. Stabilizing the rib structure, particularly the costal margin, using rib plating plays a crucial role in improving postoperative outcomes. This approach helps reduce pulmonary complications and minimizes tension in the affected area, which may lower the risk of recurrent or secondary herniation [8,9].

Chest wall and thoracoabdominal herniation can lead to severe complications, including respiratory compromise, incarceration, strangulation, and strangulation with necrosis. Loss of respiratory function or bowel perfusion constitutes a medical emergency requiring prompt surgical intervention [6,8,9]. Chest wall herniations, involving disruption of intercostal muscles and rib integrity, significantly increase the risk of respiratory failure. Severe cases can impair chest wall mechanics, leading to ventilation-perfusion mismatch, hypoventilation, and hypoxia, ultimately requiring intubation [10]. Another major concern with thoracoabdominal hernias is infection. While guidelines on preoperative antibiotic use are limited, contamination of both the thoracic and abdominal cavities must be carefully considered when planning surgical repair. In cases where the surgical field is contaminated, the use of biologic or bioprosthetic mesh is recommended for soft tissue reconstruction, as is standard practice in hernia repairs involving contaminated sites [8]. Additionally, combined transabdominal intrathoracic hernias present a particularly complex challenge, requiring a surgical approach that minimizes complications for both the abdominal and thoracic components of the hernia.

Several pathophysiological factors contribute to chest wall and diaphragmatic defects, with chronic strenuous coughing being a key risk factor. Conditions like COPD can trigger repetitive cough Valsalva maneuvers, disrupting the coordination between expiratory muscles and the abdominal wall. This imbalance leads to abnormal diaphragm and rib movement, increasing the risk of diaphragmatic injury and potential bowel loop herniation, which can impair ventilation and oxygenation [11]. In our patient with COPD, chronic cough elevated abdominal pressure, playing a significant role in his presentation. Structural chest wall defects, such as rib fractures, further compromise chest wall integrity and may lead to respiratory complications, including direct airway or lung injury [10]. Patients with COPD, asthma, osteoporosis, or advanced age are particularly susceptible to coughing-induced rib fractures, which have been linked to TDIHs [4]. Recognizing these risk factors, potential complications, and the extent of herniation is essential for guiding appropriate management and improving outcomes in similar rare clinical cases.

## Conclusions

This case underscores the importance of advanced imaging and surgical expertise in the management of rare TDIHs, particularly in patients with chronic conditions such as COPD that may exacerbate disease severity. Optimal outcomes require early CT-based identification of hernia contents, multidisciplinary collaboration, and precise operative management. Future management strategies should incorporate risk factor assessment, complication prevention, and standardized chest wall grading systems to inform surgical decision-making. While existing literature outlines treatment strategies for traumatic TDIHs, further research supporting spontaneous chest wall hernias is needed to enhance understanding and optimize future patient outcomes.
